# Ten simple rules for writing a career development award proposal

**DOI:** 10.1371/journal.pcbi.1005863

**Published:** 2017-12-14

**Authors:** Crystal M. Botham, Joshua A. Arribere, Sky W. Brubaker, Kevin T. Beier

**Affiliations:** 1 Stanford Biosciences Grant Writing Academy, Stanford University, Stanford, California, United States of America; 2 Department of Molecular, Cell, and Developmental Biology, University of California, Santa Cruz, Santa Cruz, California, United States of America; 3 Department of Microbiology and Immunology, Stanford University, Stanford, California, United States of America; 4 Department of Biology, Stanford University, Stanford, California, United States of America; 5 Department of Psychiatry and Behavioral Sciences, Stanford University, Stanford, California, United States of America; Whitehead Institute, UNITED STATES

## Introduction

Career development awards support intensive training and provide valuable funding for promising postdoctoral and/or junior faculty–level scientists during the transition to research independence. Therefore, career development awards are typically competitive and are a significant undertaking. Many countries have such awards, with the National Institutes of Health (NIH) K series being the most well known in the United States. If funded, the career development award demonstrates success in obtaining research funding, a key factor for future success. In addition, regardless of whether or not the application is funded, a well-designed proposal will help launch the transition to independence by providing a detailed plan for career development and future research. This guide provides an overview for formulating a strong career development award, with principles generalizable to other grant proposals.

## Rule 1: Give yourself time

As a guideline, we recommend that 3 months of full-time effort be dedicated to completing the application. During this writing time, you may have limited bandwidth for your research and other activities. This may seem excessive, but career development award applications often require more documents than senior-level grants. Even for experienced writers, soliciting and incorporating feedback on the various documents can be a time-consuming process. We recommend that you start on your application as early as possible and work steadily.

First, read all the instructions and create a list of all the required documents. Create a realistic timeline that outlines when you will draft, elicit feedback on, edit, and finalize the various documents. Prior applicants can be a particularly valuable resource—they can provide a sense of the required time commitment and may be willing to share their own proposal and reviewers’ comments to show you how the proposal was evaluated. Ideally, try to obtain copies of previous successful applications. For NIH K series awards, one particularly valuable resource can be the NIH Research Portfolio Online Reporting Tools, Expenditures and Results (RePORTER) tool [1]. On this site, you can read abstracts of successful grant applications. In addition, if you don’t personally have access to copies of funded grants, you can consider reaching out to postdocs at your institution that you identify through the NIH RePORTER website.

## Rule 2: Write using the review criteria as your guide

The reviewers of your application are busy. They are running labs, teaching, mentoring, writing papers and grants, working in the clinic, serving on committees, and/or balancing other personal obligations. Reviewing grants doesn’t replace these responsibilities but rather gets added on top of them. As a writer, strive to make reviewing your proposal as quick as possible by making it easy to read (see Rule 6) and easy to score.

To evaluate your application, reviewers are provided a set of criteria, which is often available to applicants. For example, the NIH K99/R00 lists the scored review criteria, including specific questions for reviewers, in the Program Announcement, Section V (Application Review Information). This section articulates the funding agency’s expectations. It is therefore essential that you provide direct, clear answers to these questions in your proposal. You can address these questions at multiple points in your proposal and emphasize your answers with unique fonts (*italics*, **bold**, or underline). Furthermore, you can use the review criteria as a source of content, structure, and language to write your proposal.

As an example, consider that the reviewer is asked the following question (emphases added): “has the candidate presented strategies to ensure a robust and unbiased approach, as appropriate for the work proposed?” In this case, you can consider including a sentence in your Research Plan that uses the same content, structure, and language; e.g., “*these multiple strategies provide a robust and unbiased approach to answer my research questions*.” Italicizing the sentence will make it stand out on the page. This parallel structure (“strategies,” “robust,” “unbiased”) makes it easy for your reviewer to locate and answer that review question. If the reviewer needs to search for or infer the information, it may negatively impact your score.

## Rule 3: Present a plan to differentiate yourself from your mentor

Reviewers for career development awards will consider whether the proposal offers a clear plan for separation from your mentor’s research. First, initiate a conversation with your mentor about which aspects of your future work you can take to your own lab. Make sure you and your advisor agree on this plan. Then, make sure your documents, as well as your mentor’s support letter, articulate a consistent plan for the mentored and independent phases of your career.

Aim to demonstrate your plan for differentiation from your mentor at multiple points throughout your proposal. For example, in your NIH “biosketch,” you can describe how the combination of your past and proposed research gives you a perspective that is unique from your mentor’s. If you learn new techniques from collaborators outside of your lab, state how this will give you a niche distinct from that of your mentor.

## Rule 4: Elicit feedback on your Specific Aims early and often

The Specific Aims document is the executive summary of your research proposal and the single most important document of your application. In fact, it is likely the first document your reviewers will read. It is therefore crucial that your Specific Aims document is clear, engaging, and exciting.

Often limited to 1 page, the Specific Aims document must succinctly convey (1) your proposal’s importance, (2) what you will do in the appropriate amount of detail, and (3) how the proposal will contribute to your training. Most of all, it is imperative that your Specific Aims document ignites the reviewers' desire to read more.

We recommend reading Yuan et al., 2016 [2] and the Specific Aims chapters in *The Grant Application Writer’s Workbook* [3] for tips on how to construct your Aims page. Then, construct a preliminary outline that includes the key information you want to convey. Ask your mentors, colleagues, and friends for feedback on the overall framework of your ideas. Seek feedback from an audience most similar to your reviewers because this will help tailor your proposal for your target audience. Next, write an early draft of your Specific Aims document and again seek feedback by requesting 3 to 4 prioritized comments from each reviewer. This will help you address the most important concerns. An iterative process of eliciting feedback and revising will greatly strengthen your writing. Getting feedback from both experts from your field (e.g., within your lab) and nonexperts (outside of lab) is important.

Once your Specific Aims document begins to take shape, engage your funding agency. Present it to the program officer (read “What to Say—and Not Say—to Program Officers” [4]) or other relevant official to check whether your proposal is suited for the agency’s funding goals.

## Rule 5: Create a Research Plan that bridges the gap between a scientific unknown and an expected payoff

Don’t fall into the common trap of simply listing the Aims and experimental details in your Research Plan. This document should describe how your project will bridge the gap between something needed and an ultimate payoff. Therefore, the need and the payoff should be clearly stated at the onset and reiterated (sparingly) throughout the document. It’s your job to convince your reviewer that your plans (Aims) are bound to successfully bridge that gap you wish to fill. You can use the following questions to help guide your Research Plan and convince a reviewer that your research is important and feasible:

1. Why is the project needed?
2. What is innovative about the project?
3. How will the project be completed?
4. How long will the project take?
5. What are the expected payoffs from the project?

Answers to the above questions correspond to the general subsections of the Research Plan, as follows: (1) Background & Significance, (2) Innovation, (3) Research Approach, (4) Timeline, and (5) Conclusions & Future Directions.

## Rule 6: Use clear writing

Clear, concise writing creates stronger and more memorable arguments. While there is a temptation to write up to the page limit of a document, a structured argument supported by only the necessary information will be more compelling.

Keep it simple. First, break down each required document into its most essential components (see Rule 2 for how to use the review criteria as your guide). Consider making bullet sentences for each point or using just a few words to state your argument. If this is not sufficiently simple, it needs to be refined. This is a great opportunity to discuss your ideas in their simplest form with mentors and colleagues.

Next, slowly build it back up. Connect the bullets with appropriate transitions so that each point flows logically to the next. Think of it as holding your reviewer’s hand as he/she reviews your documents. You must lead him/her to what needs to be done next.

Lastly, polish your writing by using sentence structures that are clear and concise. Some samples are shown in Table 1.

**Table 1:** Suggestions for how to implement clear writing.

Suggestions for clear writing	Instead of:	Write this:
**1. Limit use of the verb “to be.”**	X is an indication that Y	X indicates Y
**2. Limit prepositional phrases.**	The instrument in the lab is necessary	The lab instrument is necessary
**3. Use direct, active-voice sentences.**	Provides justification for	Justifies
**4. Avoid noun forms of verbs (nominalizations).**	The application of these techniques can	Applying these techniques can

For more information about clear writing, see Dr. Kristin Sainani’s *Writing in the Sciences* course [5].

## Rule 7: Construct a strong mentoring team

Career development awards are mentored awards. Therefore, they frequently require a dedicated team of mentors that will guide and evaluate your progress during the proposed funding period.

An appropriate mentoring team should have the necessary research qualifications, experience, scientific stature, and mentoring track record to help you enact your research and training plans. An advisory team typically consists of your primary mentor, an optional co-mentor, and 2 to 4 additional mentors. Select a team that complements your strengths. For instance, if you are proposing to use a technique new to you, find an advisor who has published using that technique. Clearly describe in your proposal how this relationship will enable you to learn the technique. Obtain letters from your mentors that affirm their commitment to mentoring you and describe, specifically, how they plan to foster your development and transition to independence. Be sure that letters from your mentors are very detailed. Include information such as how often you will meet with the mentor, how long these meetings will be, whether you will attend lab meetings, and how progress will be assessed.

As with other documents, these letters need to consider the review criteria and the funding opportunity. It is useful to meet with your mentors and advisors to ensure a mutual understanding of your research and training plan and to guarantee that they clearly articulate their roles in any provided letters. You may consider providing a template that includes the information that you wish them to convey.

## Rule 8: Design a career development plan to equip you for independence

Unlike most lab grants, career development awards include a training component. Use your career development documents to chart a path to your future success by addressing the following questions, sequentially narrowing down from big picture to specifics:

1. What are your career goals?
2. What skills do you need to achieve your career goals?
3. What activities will you engage in to attain those skills (include course numbers, meeting titles, and the names of any principal investigators [PIs] involved)?

Be thorough and rigorous: include a timeline to achieve your goals, establish a plan to monitor your progress, and obtain letters of support from additional PIs as necessary (see Rule 7).

Critically, you must articulate why additional training is necessary. If you struggle to articulate the need for additional training, you may already be equipped for your future career and may not need additional training.

## Rule 9: Use prior training and experiences to highlight your potential

Many career development awards require that you describe your background and career goals. Furthermore, you may be required to submit a document like the NIH biosketch that summarizes your prior training and experiences. Your reviewers will use these documents to determine whether you have the potential to become a successful, independent investigator based on what you have done to date and how that relates to what you propose to do. Therefore, you must demonstrate your research productivity and the quality of past training.

In these documents, instead of providing a laundry list of publications or research expertise, use elements of the classic story arc if possible. First, outline the historical background that inspired the work, including the challenge or knowledge gap addressed. Next, detail your contributions as well as the central findings and any expertise you gained. Lastly, describe the impact of your research and why it was significant.

You may also need to write a personal statement. Use this opportunity to tell a story that describes why you made key career choices and provide evidence for your long-standing and continued commitment to research. It is helpful to conclude this subsection by describing how your prior activities in conjunction with your proposed training will enable you to achieve your career goals.

## Rule 10: Weave a consistent story throughout all documents

Maintaining continuity is a very important but often underappreciated aspect of a successful grant application. All aspects of the proposal, from supplemental documents to recommendations, should mutually reinforce one another. Do not assume that saying something once is sufficient—if something is important, state it many times, in many documents. For example, if you plan to learn a technique from a member of your advisory committee, highlight this in your research strategy, sections concerning your training and career goals, as well as in letters from your mentor and the specific advisory committee member. If you state that you will visit a collaborator’s lab, you should budget related travel costs into your proposal. The more you link the different components of your research and career training plan together, the more your reviewer will feel that the proposal was thoroughly planned and well developed. Also, your reviewer will have an easier time finding relevant information (see Rule 2). Allow for time within your writing schedule to synchronize components of your proposal to tell a single, coherent story.

## Conclusion

These 10 simple rules provide a framework to construct your career development award application. The process may take a significant amount of time and effort, but it’s worth the potential payoff. Regardless of the award outcome, it will be time well spent planning your research and career. Lastly, here is the most important thing to remember: apply! You can’t win if you don’t play.
